# Teaching styles and school adjustment among migrant and non-migrant children in China: cross-sectional indirect associations via achievement goals and academic engagement

**DOI:** 10.3389/fpsyg.2026.1943367

**Published:** 2026-10-30

**Authors:** Yuqing Wan, Haoqing Feng

**Affiliations:** 1 College of Education, Zhejiang Normal University, Jinhua, China; 2 School of Marxism Studies, Zhejiang Normal University, Jinhua, China

**Keywords:** academic engagement, achievement goals, migrant children, school adjustment, teaching styles

## Abstract

This study examines the associations between teaching styles (teacher autonomy support and teacher control) and school adjustment among migrant and non-migrant children in China, with achievement goals and academic engagement modeled as potential linking variables. Cross-sectional questionnaire data were collected from 1,147 fourth- to sixth-grade students, including 527 migrant children and 620 non-migrant children. The data were analyzed using structural equation modeling with multigroup comparisons based on Wald tests. Results indicate that teacher autonomy support is significantly positively associated with school adjustment, whereas teacher control is negatively associated with it. Mastery-approach and mastery-avoidance goals, together with academic engagement, serve as key linking variables in these associations. Notably, the positive association between autonomy support and school adjustment, particularly through mastery-approach goals and direct academic engagement, is stronger for migrant children. In contrast, teacher control is negatively associated with school adjustment only in non-migrant children, linked through mastery-avoidance goals and academic engagement. These findings underscore the importance of teacher autonomy support in relation to school adjustment and suggest that educational practices could consider enhancing autonomy support and mastery-approach goals for migrant children, while reducing controlling practices for non-migrant children to improve educational outcomes.

## Introduction

Schools play a pivotal role in students’ learning and social development. Students’ ability to adapt within this environment is crucial for achieving academic goals, building positive interpersonal relationships, and sustaining overall mental and physical well-being. This adaptation process is known as school adjustment, which refers to the extent to which a child complies with school regulations and procedures, and interacts appropriately with peers and teachers without being overwhelmed by stress (Basharpoor et al., 2022). It includes five key aspects: academic adjustment, school attitude, peer relationships, teacher-student relationships, and adherence to school norms (Cui, 2008). Existing research has shown that specific aspects of positive school adjustment, such as positive teacher-student relationship, are positively related to students’ academic engagement (Gao et al., 2023). Positive school adjustment is also associated with reduced stress levels and increased life satisfaction (Chui and Chan, 2020), whereas poor school adjustment, such as negative teacher-student relationships, is associated with increased conduct problems and hyperactive behaviors (Longobardi et al., 2016), as well as heightened disengagement and underachievement (Di Lisio et al., 2025). As a comprehensive indicator of student development, school adjustment is associated with not just academic achievement but also psychological well-being and social development quality. Thus, exploring its associated factors is crucial.

According to China’s seventh national census, the country’s internal migrant population reached 376 million in 2020, including 71.09 million migrant children, nearly double the 2010 figure of 35.81 million. In this study, the term “migrant children” refers specifically to internal migrants within China rather than international immigrants, typically denoting children who relocate from rural to urban areas alongside their parents. Despite becoming an integral part of urban populations, migrant children still underperform local children in academic engagement and school adjustment. China has emphasized the importance of establishing a public service system that is based on residence, aiming to provide eligible agricultural migrants with equal rights in areas like social insurance, housing security, and compulsory education for their children. This approach seeks to ensure that these migrants have the same opportunities as local residents, thereby facilitating the urbanization of the agricultural population. Yet, migrant children’s access to local welfare services remains difficult (Ni et al., 2016), leaving them disadvantaged in urban public education and struggling with educational integration, which hinders school adjustment. Improving school adjustment for migrant children is vital not only for their academic growth but also for social integration, making research on its contributing factors highly significant.

Self-determination theory (SDT) posits that the essence of understanding human motivation lies in acknowledging the fundamental psychological needs for competence, autonomy, and relatedness (Deci and Ryan, 2000). In this context, experiencing a sense of choice and perceiving oneself as the initiator of actions can promote motivation (Bao and Lam, 2008). Research indicates that teaching styles influence students’ classroom participation and are linked to their levels of alienation from teachers and learning (Grecu et al., 2022). This relationship suggests a potential association between teaching styles and school adjustment. In this context, we explore two theoretically opposite teaching styles in line with SDT: teacher autonomy support and teacher control (Haerens et al., 2015).

Teacher autonomy support fosters mastery orientation, which in turn boosts academic achievement; conversely, teacher control predicts learned helplessness, which is associated with lower academic achievement (Filippello et al., 2020), impeding school adjustment. However, this mechanism may involve more complex cognitive-motivational processes. Students who perceive autonomy support tend to adopt mastery goals and exhibit behavioral engagement (Benita and Matos, 2021), leading to better school adjustment. Hence, examining whether achievement goals and academic engagement indirectly link teaching styles to school adjustment is essential to uncover motivation and behavior dynamics. Despite this, migrant children remain understudied, particularly regarding comparative research between migrant and non-migrant groups.

To address these issues, this study explores three key questions: (1) Do teacher autonomy support and teacher control have indirect associations with school adjustment through achievement goals and academic engagement? (2) Do these indirect associations differ between migrant and non-migrant children? (3) How do the direct relationships of teacher autonomy support and teacher control with school adjustment differ between migrant and non-migrant children? Through this exploration, the study aims to clarify the interplay among teaching styles, achievement goals, academic engagement, and school adjustment, offering insights into fostering autonomous learning experiences and adjustment skills.

### Literature review

#### Teacher autonomy support, teacher control and school adjustment

Rooted in SDT, teacher autonomy support occurs when educators adopt students’ perspectives, acknowledge their feelings, and offer informational guidance and choices, while minimizing coercive pressure (Williams and Deci, 1996). Empirical evidence has confirmed that fulfilling students’ autonomy needs not only enhances intrinsic motivation but also positively shapes academic engagement (Fu, 2019; Karimi and Sotoodeh, 2020). Specifically, perceived autonomy support fosters autonomous motivation, which subsequently promotes engagement, effort, and learning (Johansen et al., 2023), collectively contributing to improved school adjustment.

In contrast, SDT describes teacher control as a practice combining internal tactics (e.g., inducing guilt or anxiety) with external tactics (e.g., punitive directives or conditional incentives) to pressure and coerce students (De Meyer et al., 2016). Such methods correlate with maladaptive learning approaches (Vansteenkiste et al., 2005) and diminished academic achievement (Aunola and Nurmi, 2004), ultimately undermining school adjustment. Empirical evidence supports this mechanism, as controlling teaching frustrates students’ psychological needs and is demotivating (Wang et al., 2024), and has been identified as contributing to increased procrastination and reduced academic engagement (Opdenakker, 2021). Whereas prior studies have predominantly focused on non-migrant populations, this study extends the inquiry to migrant children while conducting a comparative analysis to explore variations in how teaching styles are associated with school adjustment across these groups.

#### The indirect role of academic engagement

The concept of achievement goals comprises mastery-approach, mastery-avoidance, performance-approach, and performance-avoidance goals (Elliot and McGregor, 2001). Specifically, mastery-approach goals emphasize self-improvement, mastery-avoidance goals aim to avoid incompetence relative to self-standards, performance-approach goals prioritize outperforming others, and performance-avoidance goals focus on avoiding failure in comparison to others (Van Yperen et al., 2015). Empirical evidence suggests that teaching styles play a critical role in shaping these goals (Filippello et al., 2020; Madjar et al., 2013). Teacher autonomy support fosters mastery goal orientations and enhances engagement (Benita and Matos, 2021; Filippello et al., 2020), whereas teacher control predicts performance-approach and performance-avoidance goals (Madjar et al., 2013). It is important to note that the effects of teacher support can vary depending on classroom goal structures and students’ perceptions of teaching styles (Benita and Matos, 2021). Specifically, mastery goals lead to more positive emotional experiences and behavioral engagement when they are adopted in an autonomy-supportive context or for autonomous reasons (Benita et al., 2014). In contrast, performance-avoidance goal structures may increase stress related to school performance (García-Moya et al., 2023), and controlling behavior tends to result in weaker conceptual understanding and lower engagement in deep cognitive processing (Hofferber et al., 2014).

The relationship between the different dimensions of achievement goals and school adjustment is multifaceted. For instance, Duchesne and Ratelle (2020) found that mastery-approach goals predict emotional adjustment; additionally, Yu et al. (2023) demonstrated that mastery goals correlate with resilience and well-being during educational transitions. Conversely, performance-avoidance goals correlate positively with disorganization and surface learning, while negatively with deep processing and exam performance (Elliot et al., 1999). Similarly, Remedios and Richardson (2013) summarized prior research as suggesting that mastery-avoidance goals may be associated with lower self-efficacy and academic disengagement. The studies further show distinct links between each goal dimension and teaching styles (Benita et al., 2014; Shih, 2008). Considering that SDT suggests teaching styles (autonomy support and control) are differentially correlated with the internalization of goals, and each dimension of achievement goals is associated with distinct outcomes related to school adjustment. Therefore, all four dimensions were included in the measurement model to allow for a comprehensive examination of their potential roles. Based on this rationale, we proposed an indirect pathways model within the SDT framework. We conceptualize achievement goals as the key indirect link connecting teaching styles to school adjustment. The model specifies a directional pathway wherein teaching style is correlated with the nature of goal internalization. This internalization quality is then associated with which specific achievement goal dimensions students adopt, and these adopted goal dimensions are subsequently linked to academic engagement, ultimately relating to school adjustment.

#### The indirect role of academic engagement

Empirical studies across diverse cultural contexts have validated the applicability of SDT in enhancing student motivation and engagement in learning (Perera, 2022). Academic engagement is operationalized as a metaconstruct within a three-part typology, encompassing affective, behavioral, and cognitive dimensions (Lam et al., 2012). Affective engagement involves the emotions, feelings, and reactions associated with learning activities; behavioral engagement refers to the various actions, such as effort and persistence, that students display while actively participating in the learning process; and cognitive engagement encompasses the use of meta-cognitive skills and strategies in learning (Mammadov and Schroeder, 2023).

Teacher autonomy support positively predicts various learning outcomes, such as autonomous motivation and student engagement (Mammadov and Schroeder, 2023). This support enhances students’ academic self-perception (e.g., self-efficacy, success expectancy), which strengthens behavioral and emotional engagement (Mih and Mih, 2013). Conversely, teacher control is negatively associated with students’ use of self-regulation strategies, which in turn show a positive association with academic achievement (Soenens et al., 2012). Controlling teacher behavior is also negatively associated with students’ intrinsic motivation, which in turn predicts their perception of the subject’s importance (Moreno-Murcia et al., 2018). These findings collectively emphasize the need to prioritize autonomy-supportive practices to optimize engagement. Research also indicates that higher engagement correlates with lower dropout rates, whereas disengagement predicts elevated dropout risks (Archambault et al., 2009). Thus, academic engagement is hypothesized to be associated with the relationship between teaching styles and school adjustment.

### Group differences

Studies have revealed significant disparities between migrant and non-migrant children across health and social domains (Chang et al., 2022; Dondi et al., 2020). Compared to their native peers, migrant children exhibit higher risks of malnutrition, poor physical health, mental health disorders, and mortality (Chang et al., 2022), alongside lower utilization of healthcare services (Markkula et al., 2018). These challenges are exacerbated by factors such as food insecurity, acculturation stress, and socioeconomic disadvantages, which contribute to diet-related morbidities like obesity and metabolic disorders (Dondi et al., 2020). In the Chinese context, migrant children in urban areas underperform academically relative to local students (Ma, 2020) and report heightened loneliness (Ge et al., 2024).

Besides, migrant students often struggle with emotional engagement, experiencing reduced belongingness and social exclusion (Zhang et al., 2020). Notably, migrant children often experience a decline level of school engagement in destination cities (Zhang et al., 2020), and frequent mobility further undermines classroom participation and academic performance (Gruman et al., 2008). Research emphasizes that teacher support can mitigate marginalization while boosting academic performance and school adjustment among migrant students (Zhang and Feng, 2025). Specifically, effective teaching practices, particularly those balancing academic and affective support, can mitigate these disparities by fostering intrinsic motivation and engagement in both groups (Hornstra et al., 2018; Popp et al., 2011).

It is important to recognize that disparities between migrant and non-migrant children are multifaceted. These arise from individual or family factors, like socioeconomic status and acculturative stress, as well as broader school-context factors, such as school resources, interpersonal relationships. Differences in school environments can significantly correlate with children’s academic performance and well-being. Schools that promote intercultural education and fulfilling interpersonal relationships are crucial for the integration and well-being of migrant students (Dežan and Sedmak, 2023). Additionally, school context influences students’ academic self-concept and achievement (Yang Hansen et al., 2022). Given that the current study involves separate schools for migrant and non-migrant children, it is acknowledged that group identity is confounded with potential school-level effects. In this context, the study primarily aims to examine whether the patterns of associations among key variables differ between these two groups.

To provide a theoretical basis for interpreting potential group differences in the associations of teacher control, we draw on literature that highlights the complex interplay between teacher behaviors and student outcomes. Grounded in SDT, controlling behavior is related to need frustration and increased negative affect (Liu et al., 2017), being negatively associated with students’ well-being and knowledge while being positively associated with ill-being (Behzadnia et al., 2018). At the same time, students’ responses to controlling teaching may vary across educational settings and prior experiences. For example, the perceived impact of teacher control may depend on the degree of instructional structure, the extent to which guidance is experienced as intrusive, and students’ expectations of teacher authority. Accordingly, rather than assuming that teacher control has the same effect across groups, the present study treats potential group differences as an empirical question that requires cautious interpretation.

Existing research highlights systemic disparities between migrant and non-migrant children, yet comparative studies specifically examining the relationship between teaching styles and school adjustment in these groups remain scarce. Understanding these differences is crucial for developing educational strategies that effectively support both groups. Therefore, this study aims to explore the distinct pathways through which teaching styles relate to school adjustment in these two groups.

### The current study

This study aimed to address three research questions using a multi-group structural equation model: (1) Are teacher autonomy support and teacher control indirectly associated with school adjustment through achievement goals and academic engagement? (2) Do these indirect associations differ between migrant and non-migrant children? (3) How do the direct relationships of teacher autonomy support and teacher control with school adjustment differ between migrant and non-migrant children? Drawing on SDT and the group differences outlined above, we propose three hypotheses:

H1. Teacher autonomy support is positively associated with school adjustment, whereas teacher control is negatively associated with school adjustment.

H2. Teaching styles (teacher autonomy support and teacher control) are indirectly associated with school adjustment through achievement goals and academic engagement.

H3. The model of indirect associations differs significantly between migrant and non-migrant children.

The hypothetical model is visually represented in Figure 1.

**FIGURE 1:**
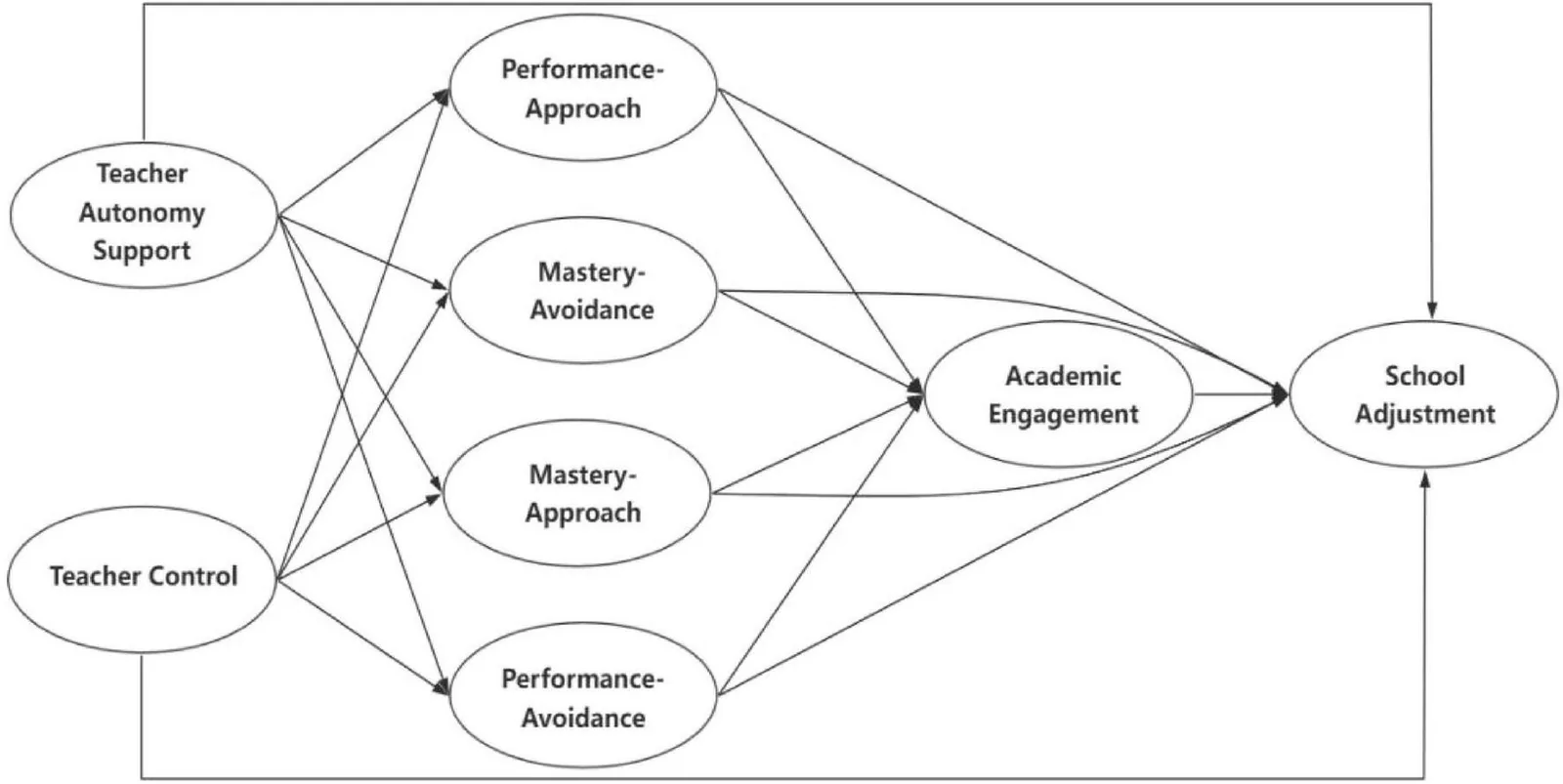
The hypothetical model.

## Materials and methods

### Participants

The study collected data between March and April 2025, focusing on students from two schools. One of these is an independent school dedicated to migrant children, who have moved with their families from China’s central and western regions to the eastern province of Zhejiang. Due to household registration constraints, these students attend specialized schools. The teachers at this independent school are specially trained to offer tailored educational guidance and support to them. The other school serves the local non-migrant children. The two schools were selected based on their distinct student demographics (migrant vs. local), with comparable class sizes and teacher-student ratios. However, we acknowledge that these two schools may still differ in unmeasured contextual characteristics, such as school climate, school resources, administrative practices, and teacher preparation. Therefore, the group comparison should be interpreted as a context-embedded comparison rather than a pure test of migration-status effects. A total of 1,147 students from grades 4 to 6 participated in the study, with 527 from the migrant school and 620 from the local school. Questionnaires were administered during class sessions under teacher supervision. Of the 1,190 surveys distributed, 1,147 valid responses were retained after excluding incomplete entries or inconsistent responses, yielding a 96.39% response rate. The participants comprised 602 boys (52.48%) and 545 girls (47.52%), with a mean age of 10.89 years (SD = 0.98). To provide a more detailed description, Table 1 presents the demographic characteristics for both the migrant and non-migrant samples.

**TABLE 1:** Demographic characteristics of migrant sample and non-migrant sample.

Demographic variable	Migrant sample (*n* = 527)	Non-migrant sample (*n* = 620)
Gender		
Male	298 (56.55%)	304 (49.03%)
Female	229 (43.45%)	316 (50.97%)
Age (mean ± SD)	10.84 ± 1.00	10.93 ± 0.95
Fathers’ education level		
Below high school	390 (74.00%)	157 (25.32%)
High school	120 (22.77%)	175 (28.23%)
College and above	17 (3.23%)	288 (46.45%)
Mothers’ education level		
Below high school	394 (74.76%)	194 (31.29%)
High school	120 (22.77%)	188 (30.32%)
College and above	13 (2.47%)	238 (38.39%)
Length of time in current city (migrant sample)	4.18 ± 1.94	–

In this study, we minimized the risk of harm to participants by ensuring that all responses were anonymized and securely stored, with access restricted to the research team only. The potential benefits of the study include providing data support for educational policy, helping teachers improve teaching methods, and increasing societal attention to children’s education. These benefits significantly outweigh the potential risks to participants. Informed consent was obtained through detailed written forms explaining the study’s purpose, procedures, and participants’ rights. We used child-friendly language to ensure understanding and guaranteed that parental consent was independent of the school agreement. Participants were informed that their participation was voluntary and that they could withdraw at any time without any consequences.

### Instruments

#### Teacher autonomy support

Teacher autonomy support was measured using an adapted version of the Learning Climate Questionnaire (Williams and Deci, 1996). Students responded to 6 items on a 5-point Likert scale ranging from “not at all true” to “completely true.” The items assessed the extent to which students perceived their teachers as providing autonomy support (e.g., “The teacher provides me with opportunities to choose.”). The Cronbach’s α coefficient was 0.936.

#### Teacher control

Teacher control was measured using an adapted version of the Controlling Teacher Scale (Jang et al., 2009). Students responded to 4 items on a 5-point Likert scale ranging from “not at all true” to “completely true.” The items assessed the extent to which students perceived their teachers as exerting control (e.g., “The teacher puts a lot of pressure on me.”). The Cronbach’s α coefficient was 0.892.

#### Achievement goal

Achievement goal was measured using an adapted version of the Motivated Strategies for Learning Questionnaire (Pintrich et al., 1993). Students responded to 12 items on a 5-point Likert scale ranging from “strongly disagree” to “strongly agree.” The items assessed four dimensions: performance-approach (e.g., “It is important for me to do better than others in my studies.”), mastery-avoidance (e.g., “I worry that I may not learn all that I ought to.”), mastery-approach (e.g., “I want to learn as much as possible.”), and performance-avoidance (e.g., “I just want to avoid doing poorly in my studies.”). The Cronbach’s α coefficients for the four dimensions were 0.823, 0.903, 0.835, and 0.750, respectively.

#### Academic engagement

Academic engagement was measured using an adapted version of the Student Engagement Questionnaire (Lam et al., 2012). Students responded to 16 items on a 5-point Likert scale ranging from “not at all true” to “completely true.” The items assessed three dimensions: behavioral engagement (e.g., “I study hard at school.”), affective engagement (e.g., “I feel good in class.”), and cognitive engagement (e.g., “I think about how the knowledge applies to real life.”). The Cronbach’s α coefficients for the three dimensions were 0.868, 0.917, and 0.917, respectively.

#### School adjustment

School adjustment was measured using an adapted version of the School Adjustment Questionnaire (Cui, 2008). Students responded to 15 items on a 5-point Likert scale ranging from “not at all true” to “completely true.” The items assessed five dimensions: academic adjustment (e.g., “Studying gives me a sense of achievement.”), school attitude (e.g., “I feel comfortable at school.”), peer relationships (e.g., “My classmates don’t like me.”), teacher-student relationships (e.g., “I avoid teachers.”), and rule compliance (e.g., “I often get punished for not following rules at school.”). The Cronbach’s α coefficients for the five dimensions were 0.617, 0.615, 0.771, 0.760, and 0.761, respectively.

#### Research procedure and data analysis

To ensure questionnaire quality, this study recruited graduate students with expertise in education as principal investigators. Prior to data collection, investigators received standardized training on survey administration protocols. After obtaining written informed consent from schools, teachers, participants, and parents, the survey was conducted in classroom settings. Investigators explained the study’s purpose and procedures to participants, provided clear instructions for questionnaire completion, and monitored the process to ensure data integrity. Students completed the questionnaires within 30 min during class, and responses were collected immediately. Ethical approval was granted by the Ethics Committee of Zhejiang Normal University.

The data were analyzed using SPSS 27.0 and Mplus 8.3, with descriptive statistics, correlation analysis, and structural equation models (SEM) serving as the primary analytical methods. Initially, Pearson correlation analysis was utilized to investigate the relationships among teaching styles, achievement goals, academic engagement, and school adjustment. Following this, SEM was applied to explore the relationships among these four variables. The Bootstrap method was employed to test the significance of the indirect roles of achievement goals and academic engagement on the relationship between teaching styles and school adjustment. Gender, age, parental education levels, and length of time in the current city (for the migrant sample) were controlled as variables to ensure the robustness of the findings. Furthermore, to prevent potential multicollinearity among variables from affecting the stability of the estimates, variance inflation factor (VIF) tests were conducted on all samples. The results were all below the commonly accepted threshold, indicating robust model stability. Lastly, multi-group analysis was performed to examine potential differences across groups: first, a measurement invariance test was conducted across groups prior to multi-group SEM comparisons. The results indicated that there was no significant degradation in model fit from configural invariance to metric invariance (ΔCFI ≤ 0.01, ΔTLI ≤ 0.01), confirming metric invariance and allowing for valid cross-group comparisons of structural paths. Second, structural equation models (SEM) were established for each group, and model fit was tested to ensure the accuracy of the analysis. Third, the Wald test was employed to compare path coefficients between the groups, determining whether significant differences existed in these paths. Fourth, if significant differences were identified, further analysis was performed to examine the direction and magnitude of these specific path differences. This analysis aimed to explore the variations in the relationships among teaching styles, achievement goals, academic engagement, and school adjustment across different groups.

## Results

### Common method bias assessment

As the data analyzed in this study were self-reported and collected at one time point, Harman’s single-factor test was employed to assess the potential for common method variance. The analysis revealed eight factors with eigenvalues greater than 1. The first factor accounted for 32.122% of the total variance, which is below the 40% threshold. These results suggest that there is no evidence of a single dominant general factor; however, because Harman’s single-factor test has well-known limitations, the possibility of common method bias cannot be ruled out completely. Therefore, the findings should be interpreted with appropriate caution.

### Descriptive statistics and correlation analysis of variables

Table 2 reveals both shared patterns and group-specific differences among the total sample, migrant children, and non-migrant children. Key commonalities include: (1) across all groups, teacher autonomy support exhibited positive correlations with performance-approach goals, mastery-approach goals, academic engagement, and school adjustment, providing support for H1; (2) in all groups, teacher control was consistently negatively associated with mastery-approach goals, academic engagement, and school adjustment, confirming H1; (3) academic engagement remained significant positive correlations with school adjustment in all groups. Notably, divergent trends emerged: (1) teacher autonomy support correlated positively with mastery-avoidance goals only in migrant children, while displaying negative correlations in non-migrant children or nonsignificant associations in the total sample; (2) teacher control showed no significant link to performance-approach goals in migrant children, contrasting with positive correlations in other samples; (3) mastery-avoidance showed a positive correlation with academic engagement for migrant children, while demonstrating a negative correlation in non-migrant children and no significant association in the total sample. Descriptively, school adjustment consistently yielded the highest means (*M* = 4.15–4.40), while teacher control showed the lowest (*M* = 1.77–1.84). Notably, the descriptive data indicate that school adjustment had mean scores approaching the scale ceiling across all samples, suggesting a potential ceiling effect.

**TABLE 2:** Means (*M*) and standard deviations (*SD*) of each variable and correlations between the variables for the total sample, migrant sample and non-migrant sample.

Sample	Variables	TAS	TC	PAp	MAv	MAp	PAv	AE	SA
Total sample	TAS	1	1	1	1	1	1		
	TC	−0.27***							
	PAp	0.24***	0.10***						
	MAv	−0.01	0.21***	0.47***					
	MAp	0.58***	−0.23***	0.47***	0.19***				
	PAv	0.16***	0.05	0.44***	0.35***	0.39***			
	AE	0.66***	−0.18***	0.34***	0.03	0.67***	0.24***		
	SA	0.51***	−0.48***	0.11***	−0.11***	0.47***	0.12***	0.55***	1
	*M*	3.97	1.81	3.47	3.23	4.13	3.89	3.9	4.28
	*SD*	1.01	1.01	1.05	1.24	0.88	0.96	0.89	0.69
Migrant sample	TAS	1	1	1	1	1	1	1	
	TC	−0.31***							
	PAp	0.27***	−0.05						
	MAv	0.22***	−0.02	0.41***					
	MAp	0.49***	−0.26***	0.53***	0.38***				
	PAv	0.24***	−0.14***	0.30***	0.22***	0.49***			
	AE	0.59***	−0.23***	0.42***	0.27***	0.62***	0.39***		
	SA	0.49***	−0.40***	0.26***	0.15***	0.48***	0.32***	0.59***	1
	*M*	3.63	1.77	3.27	3.30	3.87	3.85	3.50	4.15
	*SD*	1.00	0.82	0.92	1.01	0.87	0.86	0.85	0.64
Non-migrant sample	TAS	1	1	1	1	1	1	1	
	TC	−0.30***							
	PAp	0.13***	0.17***						
	MAv	−0.12***	0.31***	0.52***					
	MAp	0.59***	−0.25***	0.38***	0.12**				
	PAv	0.09*	0.14***	0.53***	0.43***	0.32***			
	AE	0.64***	−0.21***	0.21***	−0.08*	0.65***	0.15***		
	SA	0.49***	−0.55***	−0.03	−0.23***	0.43***	−0.01	0.50***	1
	*M*	4.26	1.84	3.64	3.17	4.36	3.93	4.23	4.40
	*SD*	0.92	1.14	1.12	1.41	0.83	1.04	0.78	0.72

TAS, teacher autonomy support; TC, teacher control; PAp, performance-approach; MAv, mastery-avoidance; MAp, mastery-approach; PAv, performance-avoidance; AE, academic engagement; SA, school adjustment; *M*, mean; *SD*, standard deviation; *n* = 1147 (total), 527 (migrant), 620 (non-migrant); *p < 0.05, **p < 0.01, ***p < 0.001.

### Structural pathway model

In this study, a structural equation model was constructed to examine the indirect pathways involving achievement goals and academic engagement, as shown in Figure 2 and Table 3. Figure 2 presents standardized direct path coefficients between adjacent variables in the model, whereas Table 3 presents the corresponding specific indirect associations. Student gender, age, parental education levels, and length of time in the current city were included as control variables. The results indicated that the model fit well: χ^2^/df = 4.538 (χ^2^ = 2255.420, df = 497, RMSEA = 0.056, CFI = 0.926, TLI = 0.913, SRMR = 0.080). The analysis revealed several significant pathways. Teacher autonomy support was found to have a significant positive indirect association with school adjustment through mastery-approach goals and academic engagement, thus supporting H2. However, it also had a significant negative indirect association with school adjustment, with mastery-avoidance goals and academic engagement forming a sequential link. Additionally, teacher autonomy support was positively associated with school adjustment through academic engagement. Conversely, teacher control demonstrated a significant negative indirect association with school adjustment through the sequential indirect pathway involving mastery-avoidance goals and academic engagement, as well as through mastery-approach goals and academic engagement. These findings further support H2.

**FIGURE 2:**
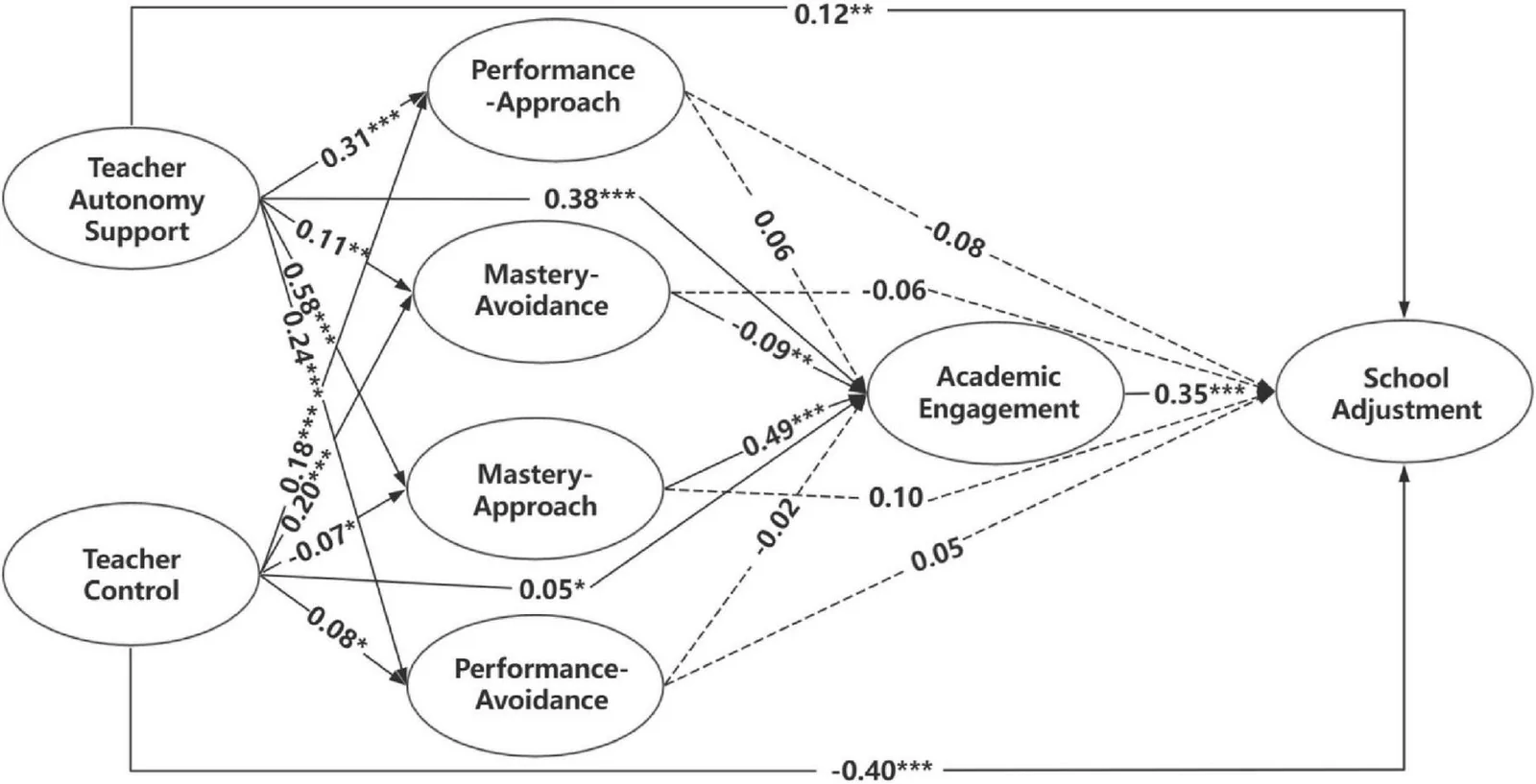
Standardized direct path coefficients for the total sample. *p < 0.05, **p < 0.01, ***p < 0.001.

**TABLE 3:** Standardized specific indirect effects for the total sample.

Pathway	Estimate	SE	*p*
TM1-AG1-LE-SA	0.007	0.004	0.131
TM1-AG2-LE-SA	−0.004	0.002	0.033
TM1-AG3-LE-SA	0.100	0.017	0.000
TM1-AG4-LE-SA	−0.001	0.003	0.641
TM1-AG1-SA	−0.024	0.014	0.083
TM1-AG2-SA	−0.006	0.004	0.134
TM1-AG3-SA	0.057	0.032	0.075
TM1-AG4-SA	0.012	0.010	0.219
TM1-LE-SA	0.135	0.021	0.000
TM2-AG1-LE-SA	0.004	0.003	0.141
TM2-AG2-LE-SA	−0.006	0.003	0.010
TM2-AG3-LE-SA	−0.012	0.006	0.025
TM2-AG4-LE-SA	0.000	0.001	0.647
TM2-AG1-SA	−0.014	0.008	0.090
TM2-AG2-SA	−0.011	0.007	0.102
TM2-AG3-SA	−0.007	0.005	0.143
TM2-AG4-SA	0.004	0.004	0.281
TM2-LE-SA	0.018	0.009	0.060

TM1, teacher autonomy support; TM2; teacher control; AG1, performance-approach; AG2, mastery-avoidance; AG3, mastery-approach; AG4, performance-avoidance; AE, academic engagement; SA, school adjustment. The same notation applies below.

### Differences between migrant and non-migrant children

Structural model testing for both migrant and non-migrant children revealed differences in certain indirect pathways linking teacher autonomy support and teacher control to school adjustment. For migrant children, the model demonstrated a good fit: χ^2^/df = 2.529 (χ^2^ = 1256.883, df = 497), RMSEA = 0.054, CFI = 0.911, TLI = 0.896, SRMR = 0.060, as illustrated in Figure 3 and Table 4. In this group, teacher autonomy support was positively associated with school adjustment through mastery-approach goals and academic engagement. Furthermore, teacher autonomy support also had a significant positive association with school adjustment via academic engagement.

**FIGURE 3:**
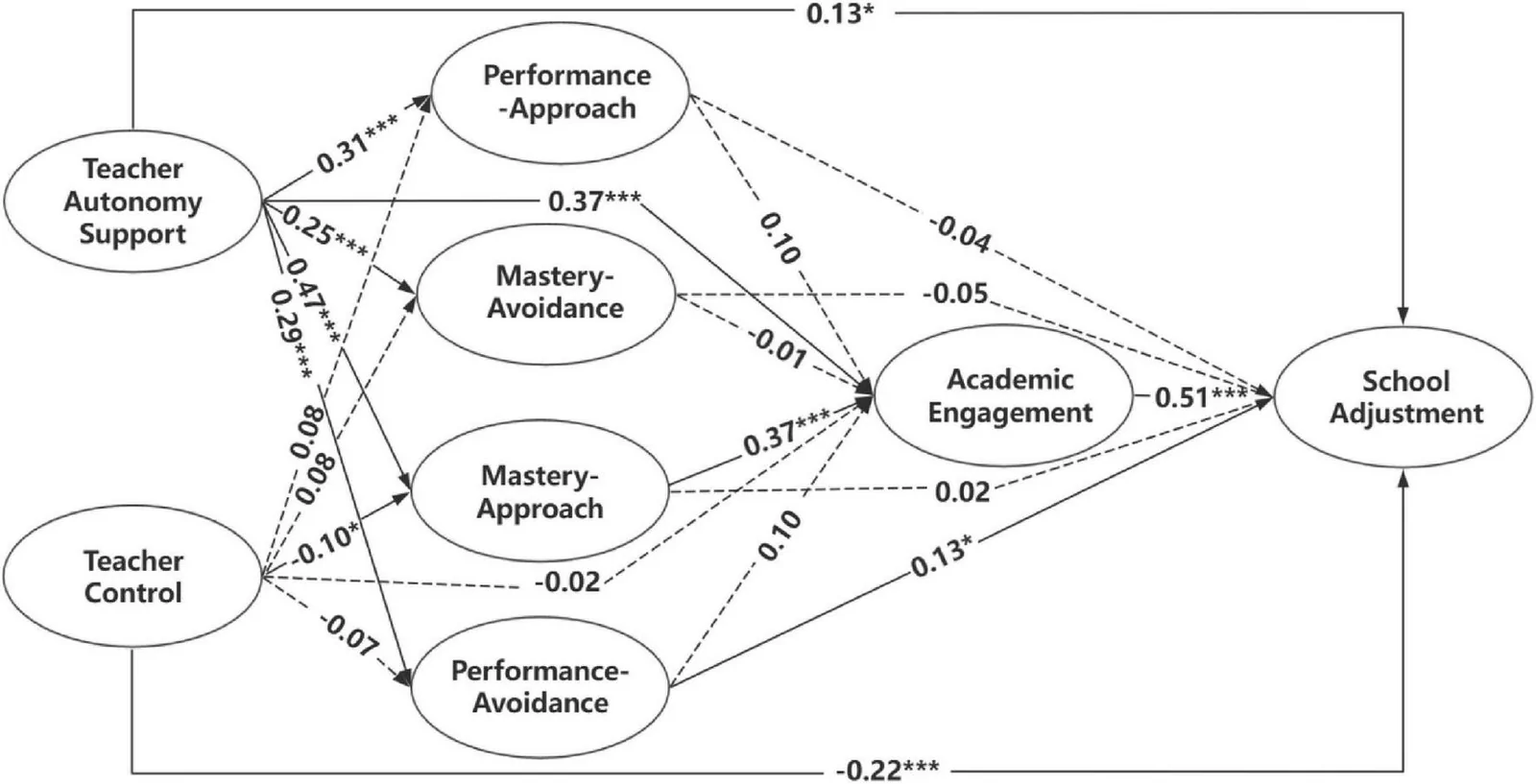
Standardized direct path coefficients for the migrant children sample. *p < 0.05, **p < 0.01, ***p < 0.001.

**TABLE 4:** Standardized specific indirect effects for the migrant children sample.

Pathway	Estimate	SE	*p*
TM1-AG1-LE-SA	0.015	0.010	0.139
TM1-AG2-LE-SA	−0.001	0.006	0.816
TM1-AG3-LE-SA	0.089	0.025	0.000
TM1-AG4-LE-SA	0.015	0.010	0.139
TM1-AG1-SA	−0.012	0.021	0.560
TM1-AG2-SA	−0.013	0.012	0.285
TM1-AG3-SA	0.011	0.042	0.803
TM1-AG4-SA	0.039	0.020	0.057
TM1-LE-SA	0.190	0.033	0.000
TM2-AG1-LE-SA	0.004	0.004	0.302
TM2-AG2-LE-SA	0.000	0.002	0.818
TM2-AG3-LE-SA	−0.020	0.011	0.069
TM2-AG4-LE-SA	−0.004	0.004	0.331
TM2-AG1-SA	−0.003	0.006	0.584
TM2-AG2-SA	−0.004	0.005	0.364
TM2-AG3-SA	−0.002	0.009	0.804
TM2-AG4-SA	−0.010	0.009	0.286
TM2-LE-SA	−0.010	0.021	0.640

Meanwhile, for non-migrant children, the model fit well: χ^2^/df = 3.341 (χ^2^ = 1580.241, df = 473), RMSEA = 0.061, CFI = 0.924, TLI = 0.911, SRMR = 0.096, as shown in Figure 4 and Table 5. Teacher autonomy support had a significant positive association with school adjustment through mastery-approach goals and academic engagement, and was also positively related to school adjustment through academic engagement. However, teacher control was negatively associated with school adjustment, with mastery-avoidance goals and academic engagement serving as sequential linking variables.

**FIGURE 4:**
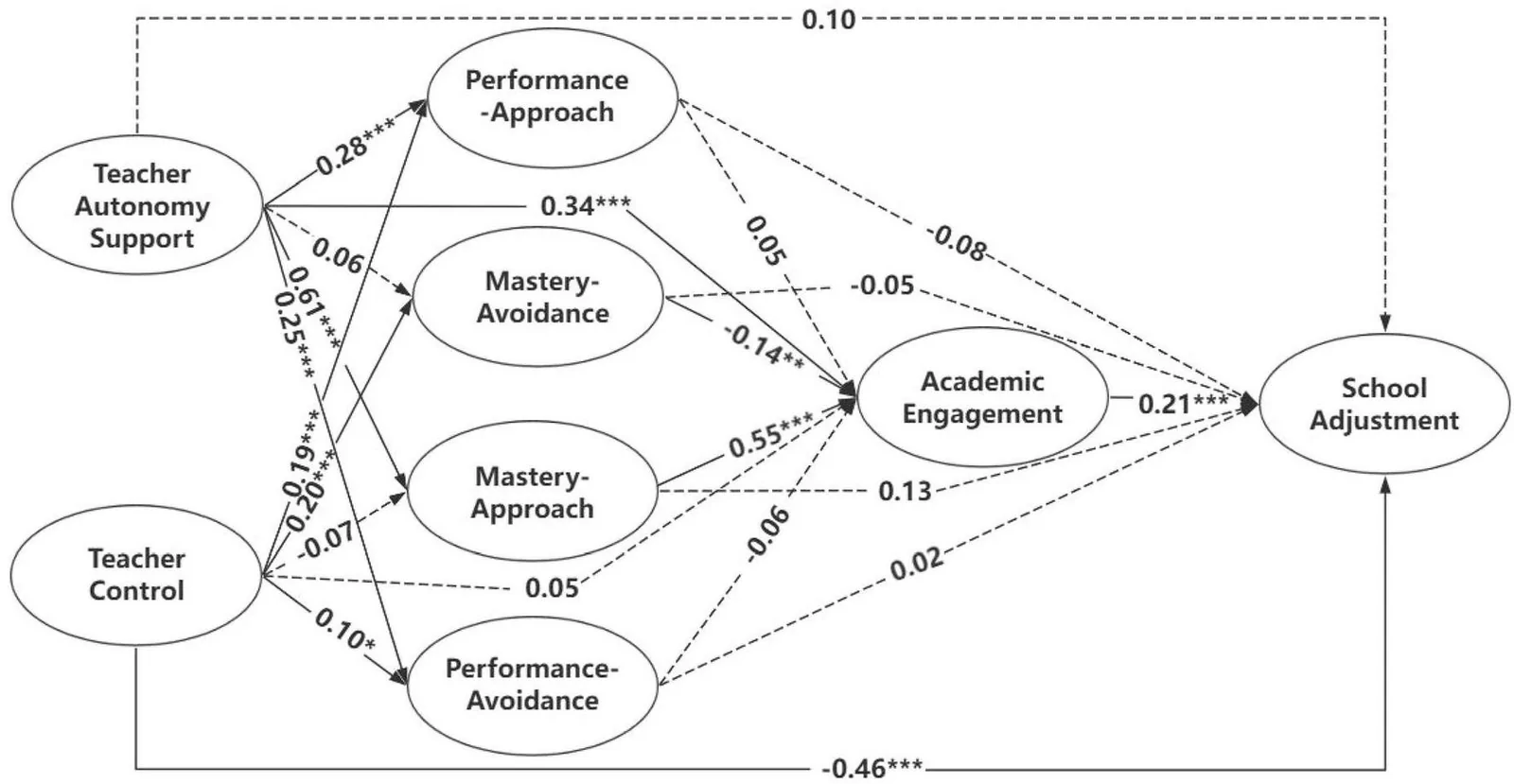
Standardized direct path coefficients for the non-migrant children sample. *p < 0.05, **p < 0.01, ***p < 0.001.

**TABLE 5:** Standardized specific indirect effects for the non-migrant children sample.

Pathway	Estimate	SE	*p*
TM1-AG1-LE-SA	0.003	0.003	0.395
TM1-AG2-LE-SA	−0.002	0.002	0.224
TM1-AG3-LE-SA	0.071	0.022	0.001
TM1-AG4-LE-SA	−0.003	0.003	0.317
TM1-AG1-SA	−0.023	0.018	0.188
TM1-AG2-SA	−0.003	0.003	0.434
TM1-AG3-SA	0.081	0.045	0.074
TM1-AG4-SA	0.006	0.015	0.682
TM1-LE-SA	0.074	0.023	0.001
TM2-AG1-LE-SA	0.002	0.002	0.402
TM2-AG2-LE-SA	−0.006	0.003	0.033
TM2-AG3-LE-SA	−0.009	0.006	0.120
TM2-AG4-LE-SA	−0.001	0.001	0.365
TM2-AG1-SA	−0.015	0.012	0.199
TM2-AG2-SA	−0.009	0.010	0.356
TM2-AG3-SA	−0.010	0.008	0.198
TM2-AG4-SA	0.002	0.006	0.689
TM2-LE-SA	0.011	0.009	0.192

### Wald test

A multigroup structural equation modeling analysis was conducted to systematically examine the differences in pathway relationships between migrant and non-migrant children. The focus was on how teacher autonomy support and teacher control are linked to school adjustment. The Wald test results (see Table 6) revealed significant group differences (*p* < 0.05) in 14 out of 18 potential pathways. Notably, two core paths were statistically significant in both migrant and non-migrant groups: Teacher autonomy support positively relates to school adjustment through mastery-approach goals and academic engagement, and also directly through academic engagement.

**TABLE 6:** Wald tests for indirect effects.

Model test	Wald	df	*p*
TM1-AG1-LE-SA	7.474	1	0.006
TM1-AG2-LE-SA	6.397	1	0.011
TM1-AG3-LE-SA	13.812	1	0.000
TM1-AG4-LE-SA	12.113	1	0.001
TM1-AG1-SA	9.623	1	0.002
TM1-AG2-SA	0.207	1	0.649
TM1-AG3-SA	12.747	1	0.000
TM1-AG4-SA	4.513	1	0.034
TM1-LE-SA	17.509	1	0.000
TM2-AG1-LE-SA	7.307	1	0.007
TM2-AG2-LE-SA	5.874	1	0.015
TM2-AG3-LE-SA	16.068	1	0.000
TM2-AG4-LE-SA	7.773	1	0.005
TM2-AG1-SA	7.416	1	0.007
TM2-AG2-SA	2.212	1	0.137
TM2-AG3-SA	2.853	1	0.091
TM2-AG4-SA	3.616	1	0.057
TM2-LE-SA	15.268	1	0.000

TM1, teacher autonomy support; TM2, teacher control; AG1, performance-approach; AG2, mastery-avoidance; AG3, mastery-approach; AG4, performance-avoidance; AE, academic engagement; SA, school adjustment.

Further analysis indicated differences in indirect pathways between the two groups. For teacher autonomy support relationships, the strength of the pathway from teacher autonomy support through mastery-approach goals and academic engagement to school adjustment was significantly stronger for migrant children compared to non-migrant children, confirming H3. Similarly, the strength of the pathway from teacher autonomy support through academic engagement to school adjustment was stronger for migrant children than for non-migrant children. In contrast, for teacher control relationships, the pathway from teacher control through mastery-avoidance goals and academic engagement to school adjustment exhibited a significant negative association exclusively in non-migrant children, with no significant association in migrant children, further validating H3.

## Discussion

This study examines the cross-sectional indirect associations between two distinct teaching styles and school adjustment, revealing three key findings. First, the observed associations suggest that achievement goals (mastery-approach and mastery-avoidance) and academic engagement function as potential chain links connecting teaching styles and school adjustment. Second, teacher autonomy support exhibited a stronger positive association with school adjustment for migrant children compared to their non-migrant peers, particularly via pathways involving mastery-approach goals and direct academic engagement. Third, it was observed that teacher control was negatively linked to non-migrant children’s school adjustment through mastery-avoidance goals and academic engagement, with no significant association for migrant children. Because the present study is cross-sectional, these findings should be interpreted as evidence of indirect associations rather than causal mediation or temporal ordering.

### The indirect roles of achievement goals and academic engagement

The results indicated indirect associations between teaching styles and school adjustment involving achievement goals and academic engagement. Specifically, teacher autonomy support is significantly associated with school adjustment through three pathways: First, teacher autonomy support is positively associated with school adjustment through academic engagement. Consistent with the findings of Jang et al. (2012), students’ perception of autonomy support affects their satisfaction of autonomy needs, which in turn influences their engagement and ultimately impacts academic outcomes. This observation also aligns with the SDT perspective, as discussed by Ryan and Deci (2020), which suggests that promoting psychological wellness through autonomy support leads to improved educational outcomes. Our study further clarifies this relationship by demonstrating that when students perceive autonomy support, they are more likely to engage with their academic tasks, thereby facilitating their adjustment to school environments. This pathway highlights the essential role of autonomy support in fostering an engaging and adaptive educational experience.

Second, teacher autonomy support is positively associated with school adjustment through mastery-approach goals and academic engagement. Consistent with the findings of Ciani et al. (2011), who found that having more self-determined motives related to the class predicted mastery-approach goals. This observation also aligns with Benita and Matos (2021), who discovered that when students perceive teachers as supportive of mastery goals, they are more likely to endorse these goals and engage behaviorally. Our study further illuminates this relationship by demonstrating that when students perceive teacher autonomy support, they are more inclined to pursue mastery-approach goals and increase their academic engagement, thereby facilitating their adjustment to school environments. This pathway underscores the crucial role of teachers in supporting students’ mastery goals and enhancing both their academic engagement and positive school adjustment.

Third, however, teacher autonomy support is also negatively associated with school adjustment via the sequential indirect associations of mastery-avoidance goals and academic engagement. Although a large volume of research has confirmed that teacher support is conducive to students’ academic motivation, engagement, and performance in school (Federici and Skaalvik, 2014; Patrick et al., 2007), its effects can vary depending on classroom goal structures and students’ perceptions of teaching styles (Benita and Matos, 2021). Specifically, García-Moya et al. (2023) suggest that performance-avoidance goal structures may increase stress of school performance. Consequently, this avoidance behavior directly diminishes students’ academic effort and participation, thereby hindering their school adjustment. Overall, these pathways highlight the complex role of teacher autonomy support in relating to school adjustment. While it primarily serves as a positive force through mastery-approach motivation and direct engagement, educators could also be vigilant about potential unintended consequences associated with avoidance-related goal adoption.

In contrast, the study reveals that teacher control is negatively associated with school adjustment through two distinct yet interrelated pathways: first, teacher control is negatively related to school adjustment through mastery-avoidance goals and academic engagement. Specifically, consistent with SDT, a controlling teaching style heightens students’ fear of failure and avoidance tendencies, as they perceive academic tasks as externally imposed rather than self-directed. This avoidance orientation reduces behavioral and cognitive engagement, ultimately impairing school adjustment. This is consistent with Remedios and Richardson (2013), who summarized prior research suggesting that mastery-avoidance goals may be associated with lower self-efficacy and academic disengagement. Further empirical evidence supports this mechanism, as demonstrated by Wang et al. (2024), who found that controlling teaching frustrates students’ psychological needs and is demotivating. Similarly, Opdenakker (2021) identified controlling teaching styles as contributing to increased procrastination and reduced academic engagement. Thus, when students prioritize avoiding incompetence over genuine learning, their academic participation diminishes, exacerbating adjustment difficulties.

Second, teacher control is also significantly negatively associated with school adjustment through mastery-approach goals and academic engagement. While mastery-approach goals typically promote adaptive outcomes, controlling environments may distort their motivational benefits. This is consistent with the findings of Moreno-Murcia et al. (2018), which indicate that controlling teacher behavior is negatively associated with students’ intrinsic motivation, which in turn predicts their perception of the subject’s importance. Here, controlling teaching, such as punishments, pressuring rewards, undermines students’ psychological needs, leading to controlled motivation and lower performance (De Meyer et al., 2014). Consequently, though students may adopt approach-oriented goals, their engagement remains superficial, as their motivation stems from compliance rather than authentic interest.

### Differences in indirect pathways between migrant and non-migrant children

The study reveals three notable differences in how teaching styles relate to school adjustment between migrant and non-migrant children. First, analyses of the indirect pathways involving mastery-approach goals and academic engagement reveal that teacher autonomy support is more strongly associated with school adjustment for migrant children than for non-migrant children. A plausible explanation is that migrant children may respond differently to clear structure and informative guidance when navigating unfamiliar academic settings. Unlike coercive teacher control, such autonomy-supportive guidance provides clarity while preserving students’ sense of volition. When autonomy-supportive teaching is paired with well-defined goals, migrant children may develop more effective learning frameworks that enhance adaptability, aligning with Zhang and Feng (2025) observation that teacher support mitigates marginalization while boosting academic performance and school adjustment among migrant students. This combination may be a key factor for migrant children in establishing clear academic objectives and improving engagement, both essential for their school adaptation.

Second, the observed differences in the indirect role of academic engagement indicate that the association between teacher autonomy support and school adjustment through this pathway is more pronounced for migrant children than for non-migrant children. This disparity might be related to the different role of academic engagement for migrant children, as noted by Liu et al. (2019), who emphasized that teacher support is directly linked to migrant children’s academic achievement. Furthermore, migrant children often face unique challenges such as cultural differences, and disruptions in their educational trajectories, making the role of teacher autonomy support particularly critical. By fostering a supportive and engaging learning environment, teachers can help mitigate these challenges, encouraging migrant children to set clear academic goals and remain actively engaged in their education. This holistic approach not only enhances their academic performance but also facilitates their overall adjustment and integration into the school community. While this provides a plausible interpretation, it requires further empirical validation.

Third, the indirect roles of mastery-avoidance goals and academic engagement in the relationship between teacher control and school adjustment differ significantly between migrant and non-migrant children, with a significant negative association observed in non-migrant children, while this association is not significant for migrant children. This indicates that the hypothesized mechanism operates differently across groups. The significant negative pathway observed among non-migrant children aligns with existing literature, controlling teaching styles may undermine students’ positive functioning and engagement, leading to poor student outcomes (Reeve, 2009), thereby hindering their school adjustment. In contrast, the non-significant association for migrant children should be interpreted cautiously. One possible explanation is that migrant children may perceive teacher control differently depending on the local educational context, prior schooling experiences, or expectations regarding teacher authority. However, the present study did not directly measure these contextual or psychological factors, so this interpretation remains tentative. Therefore, rather than concluding that teacher control is non-problematic for migrant children, it is more appropriate to state that the current data do not show a significant indirect association in this group, and that further research is needed to identify the conditions under which controlling teaching may or may not undermine engagement and school adjustment.

## Implications

### Theoretical implications

This study, grounded in SDT, advances three theoretical insights of how teaching styles relate to school adjustment through distinct motivational and behavioral pathways. First, findings from this specific context provide support consistent with SDT’s core principles. This suggests that teacher autonomy support may be positively linked to school adjustment through the sequential indirect associations of mastery-approach goals and academic engagement. In contrast, controlling teaching styles are associated with higher levels of maladjustment via mastery-avoidance goals and academic engagement. This finding provides empirical support for the core idea of SDT, which is that autonomy support enhances adaptive motivation and behavior by satisfying the basic psychological needs of competence, autonomy, and relatedness, whereas controlling teaching methods lead to maladjustment by preventing the satisfaction of these needs.

Second, the study enriches the literature by revealing the indirect role of academic engagement, in bridging teaching practices to adjustment outcomes. Consistent with SDT, which posits that autonomy-supportive environments foster internalization and engagement, our finding highlights academic engagement as a potential key mediator in the association between teacher autonomy support and school adjustment. This extends previous research by Jian (2022), which indicates that self-efficacy and academic motivation positively influence sustained engagement and achievement.

Third, the study identifies significant differences in the responses of migrant and non-migrant children to teacher autonomy support and teacher control. Migrant children tend to be more responsive to teacher support, whereas non-migrant children are more negatively associated with teacher control. This finding suggests potential benefits of teachers considering the diverse backgrounds of students when designing instructional strategies. By doing so, they could better address the motivational and educational needs of various student groups, thereby enhancing educational outcomes. Additionally, this study provides a theoretical foundation for developing more personalized support and management strategies in educational practice, facilitating greater opportunities for students to receive the guidance they need to succeed academically and enhance their overall well-being and school adjustment.

### Practical implications

The research findings provide practical insights for improving school adjustment among both migrant and non-migrant children. Differentiated strategies could be considered in areas such as teaching practices, home-school collaboration, and teacher professional development. In teaching practice, teacher autonomy support could be prioritized, particularly through strategies tailored to each group’s motivational profile. For migrant children, practitioners might consider emphasizing mastery-approach goals by incorporating task choice, process-oriented feedback, and opportunities for self-directed learning. For non-migrant children, it is beneficial to minimize controlling teaching behaviors by avoiding punitive directives and excessive performance pressure, and instead adopting needs-supportive strategies in line with SDT. This approach may be associated with lower avoidance tendencies and stronger intrinsic motivation.

Home-school collaboration could be oriented toward building a systemic support network to reinforce autonomy-supportive practices across settings. Schools could organize workshops for both teachers and parents, focusing on strategies such as growth-oriented communication that emphasizes effort-based praise and collaborative problem-solving. Such alignment between home and school environments enhances the benefits of autonomy support, particularly for migrant children who may lack external resources. Regular dialogue between educators and parents could be encouraged as a potential approach to tailor support, with attention to group-specific challenges such as acculturation stress or social integration barriers.

For teacher professional development, training programs might consider integrating sensitivity modules on group differences. These modules could help equip teachers to recognize and adapt to the distinct needs of migrant and non-migrant students. Dynamic assessment tools might be used as a complement by providing real-time data on students’ goal orientations and engagement levels, potentially enabling teachers to deliver targeted interventions. Also, training programs could incorporate regular feedback and ongoing professional support. This inclusion could assist teachers continuously enhancing their teaching practices and in addressing the diverse needs of the students more effectively. These recommendations collectively suggest the potential benefits of contextually adapting teaching styles, enhancing support systems, and refining teacher competencies to support school adjustment through different pathways for migrant and non-migrant children. However, causal interpretations require verification via longitudinal designs.

## Limitations and future research

This study presents several limitations that should be acknowledged, along with suggestions for future research. One major methodological limitation concerns the school-based sampling design. Because migrant and non-migrant children were recruited from different schools, migration status is confounded with school-level characteristics such as school climate, leadership, resources, teacher preparation, and institutional practices. As a result, the observed group differences cannot be interpreted as migration-status effects alone. Future research should address this by integrating both student populations within the same schools to control for contextual factors, or by using multi-school, matched-school, or multilevel designs to disentangle student-level and school-level influences. Furthermore, the measurement approach has its limitations. The student questionnaires assessed teacher styles in a generalized manner without differentiating subjects, which may undermine credibility. Individual differences in perceptions and adjustment were also not fully accounted for. Future research should address these limitations by differentiating subjects and controlling for individual variables. Incorporating multi-source data (e.g., teacher reports, observations) is recommended to reduce self-report bias. Theoretical and analytical constraints are also evident. The cross-sectional design precludes causal inferences and does not support temporal or causal mediation interpretations. Although the present analyses identify statistically significant indirect associations, they cannot establish the directionality or sequence of effects assumed in the proposed chain mediation model. Longitudinal or experimental designs will be necessary to test whether the observed relationships unfold in the hypothesized order over time. Additionally, the failure to model the nested structure of students within schools and to address potential ceiling effects in some variables limits the robustness of the conclusions. Future research should employ longitudinal or natural experimental designs, utilize hierarchical linear modeling, and conduct more nuanced data diagnostics. Finally, regarding sample representativeness, the data were limited to the Zhejiang region. Extending the study to other regions and critical developmental stages, such as adolescence, would enhance generalizability.

## Data Availability

To protect the privacy and confidentiality of the participants, the datasets used and/or analyzed during the current study are available from YW on reasonable request.

## References

[B1] Archambault I. Janosz M. Morizot J. Pagani L. (2009). Adolescent behavioral, affective, and cognitive engagement in school: Relationship to dropout. *J. Sch. Health* 79, 408–415. 10.1111/j.1746-1561.2009.00428.x 19691715

[B2] Aunola K. Nurmi J. E. (2004). Maternal affection moderates the impact of psychological control on a child’s mathematical performance. *Dev. Psychol.* 40, 965–978. 10.1037/0012-1649.40.6.965 15535751

[B3] Bao X. H. Lam S. F. (2008). Who makes the choice? Rethinking the role of autonomy and relatedness in Chinese children’s motivation. *Child Dev.* 79, 269–283. 10.1111/j.1467-8624.2007.01125.x 18366423

[B4] Basharpoor S. Heidari F. Narimani M. Barahmand U. (2022). School adjustment, engagement and academic self-concept: Family, child, and school factors. *J. Psychol. Counsellors Sch.* 32, 23–38. 10.1017/jgc.2020.6

[B5] Behzadnia B. Adachi P. J. Deci E. L. Mohammadzadeh H. (2018). Associations between students’perceptions of physical education teachers’interpersonal styles and students’wellness, knowledge, performance, and intentions to persist at physical activity: A self-determination theory approach. *Psychol. Sport Exerc.* 39, 10–19. 10.1016/j.psychsport.2018.07.003

[B6] Benita M. Matos L. (2021). Internalization of mastery goals: The differential effect of teachers’. autonomy support and control. *Front. Psychol.* 11:599303. 10.3389/fpsyg.2020.599303 33613356 PMC7892473

[B7] Benita M. Roth G. Deci E. L. (2014). When are mastery goals more adaptive? It depends on experiences of autonomy support and autonomy. *J. Educ. Psychol.* 106, 258–267. 10.1037/a0034007

[B8] Chang R. Li C. Qi H. Zhang Y. Zhang J. (2022). Birth and health outcomes of children migrating with parents: A systematic review and meta-analysis. *Front. Pediatr.* 10:810150. 10.3389/fped.2022.810150 35911841 PMC9326113

[B9] Chui R. C. Chan C. (2020). Positive thinking, school adjustment and psychological well-being among chinese college students. *Open Psychol. J.* 13, 151–159. 10.2174/1874350102013010151

[B10] Ciani K. D. Sheldon K. M. Hilpert J. C. Easter M. A. (2011). Antecedents and trajectories of achievement goals: A self-determination theory perspective. *Br. J. Educ. Psychol.* 81, 223–243. 10.1348/000709910X517399 21542816

[B11] Cui N. (2008). *A Study on the Relationship of School Adaptation and Self-Conception Among Junior School Students.* Master’s thesis, El Paso, TX: Southwest University.

[B12] De Meyer J. Soenens B. Aelterman N. De Bourdeaudhuij I. Haerens L. (2016). The different faces of controlling teaching: Implications of a distinction between externally and internally controlling teaching for students’ motivation in physical education. *Phys. Educ. Sport Pedagogy* 21, 632–652. 10.1080/17408989.2015.1112777

[B13] De Meyer J. Tallir I. B. Soenens B. Vansteenkiste M. Aelterman N. Van den Berghe L. et al. (2014). Does observed controlling teaching behavior relate to students’ motivation in physical education? *J. Educ. Psychol.* 106, 541–554. 10.1037/a0034399

[B14] Deci E. L. Ryan R. M. (2000). The “what” and “why” of goal pursuits: Human needs and the self-determination of behavior. *Psychol. Inquiry* 11, 227–268. 10.1207/s15327965pli1104_01

[B15] Dežan L. Sedmak M. (2023). How do you feel at school? A cross-country comparative analysis of migrant adolescents’ school well-being. *Front. Educ.* 8:1003762. 10.3389/feduc.2023.1003762

[B16] Di Lisio G. Milá Roa A. Halty A. Berástegui A. Couso Losada A. Pitillas C. (2025). Nurturing bonds that empower learning: A systematic review of the significance of teacher-student relationship in education. *Front. Educ.* 10:1522997. 10.3389/feduc.2025.1522997

[B17] Dondi A. Piccinno V. Morigi F. Sureshkumar S. Gori D. Lanari M. (2020). Food insecurity and major diet-related morbidities in migrating children: A systematic review. *Nutrients* 12:379. 10.3390/nu12020379 32023929 PMC7071308

[B18] Duchesne S. Ratelle C. F. (2020). Achievement goals, motivations, and social and emotional adjustment in high school: A longitudinal mediation test. *Educ. Psychol.* 40, 1033–1051. 10.1080/01443410.2020.1778641

[B19] Elliot A. J. McGregor H. A. (2001). A 2 X 2 achievement goal framework. *J. Pers. Soc. Psychol.* 80, 501–519. 10.1037/0022-3514.80.3.501 11300582

[B20] Elliot A. J. McGregor H. A. Gable S. (1999). Achievement goals, study strategies, and exam performance: A mediational analysis. *J. Educ. Psychol.* 91, 549–563. 10.1037/0022-0663.91.3.549

[B21] Federici R. A. Skaalvik E. M. (2014). Students’ perceptions of emotional and instrumental teacher support: Relations with motivational and emotional responses. *Intern. Educ. Stud.* 7:21. 10.5539/ies.v7n1p21

[B22] Filippello P. Buzzai C. Costa S. Orecchio S. Sorrenti L. (2020). Teaching style and academic achievement: The mediating role of learned helplessness and mastery orientation. *Psychol. Sch.* 57, 5–16. 10.1002/pits.22315 33136418

[B23] Fu D. (2019). “The Influence of Autonomy-Supportive Teaching Environment on Students’ Academic Performance,” in *Proceedings of the 2019 AERA Annual Meeting. 2019 AERA Annual Meeting*, Toronto. 10.3102/1437089

[B24] Gao Q. Bao C. Du H. Yan R. (2023). The mediating role of basic psychological needs satisfaction in the relationship between teacher-student relationships and academic engagement in china. *Asia Pacific J. Educ.* 43, 514–525. 10.1080/02188791.2021.1933380

[B25] García-Moya I. Díez M. Paniagua C. (2023). Stress of school performance among secondary students: The role of classroom goal structures and teacher support. *J. Sch. Psychol.* 99:101222. 10.1016/j.jsp.2023.05.005 37507190

[B26] Ge L. F. Wu R. Y. Zhong B. L. (2024). Elevated levels of loneliness in migrant children compared to nonmigrant children in urban China: A systematic review and meta-analysis of comparative studies. *Curr. Opin. Psychiatry* 37, 162–171. 10.1097/YCO.0000000000000933 38415713

[B27] Grecu A. L. Hadjar A. Simoes Loureiro K. (2022). The role of teaching styles in the development of school alienation and behavioral consequences: A mixed methods study of luxembourgish primary schools. *Sage Open* 12:21582440221105477. 10.1177/21582440221105477

[B28] Gruman D. H. Harachi T. W. Abbott R. D. Catalano R. F. Fleming C. B. (2008). Longitudinal effects of student mobility on three dimensions of elementary school engagement. *Child Dev.* 79, 1833–1852. 10.1111/j.1467-8624.2008.01229.x 19037953 PMC3870003

[B29] Haerens L. Aelterman N. Vansteenkiste M. Soenens B. Van Petegem S. (2015). Do perceived autonomy-supportive and controlling teaching relate to physical education students’motivational experiences through unique pathways? Distinguishing between the bright and dark side of motivation. *Psychol. Sport Exerc.* 16, 26–36. 10.1016/j.psychsport.2014.08.013

[B30] Hofferber N. Eckes A. Wilde M. (2014). Effects of autonomy supportive vs. controlling teachers’ behavior on students’ achievement. *Eur. J. Educ. Res.* 3, 177–184. 10.12973/eu-jer.3.4.177

[B31] Hornstra L. Stroet K. van Eijden E. Goudsblom J. Roskamp C. (2018). Teacher expectation effects on need-supportive teaching, student motivation, and engagement: A self-determination perspective. *Educ. Res. Eval.* 24, 324–345. 10.1080/13803611.2018.1550841

[B32] Jang H. Kim E. J. Reeve J. (2012). Longitudinal test of self-determination theory’s motivation mediation model in a naturally occurring classroom context. *J. Educ. Psychol.* 104, 1175–1188. 10.1037/a0028089

[B33] Jang H. Reeve J. Ryan R. M. Kim A. (2009). Can self-determination theory explain what underlies the productive, satisfying learning experiences of collectivistically oriented korean students? *J. Educ. Psychol.* 101, 644–661. 10.1037/a0014241

[B34] Jian Z. (2022). Sustainable engagement and academic achievement under impact of academic self-efficacy through mediation of learning agility-evidence from music education students. *Front. Psychol.* 13:899706. 10.3389/fpsyg.2022.899706 35774941 PMC9237460

[B35] Johansen M. O. Eliassen S. Jeno L. M. (2023). The bright and dark side of autonomy: How autonomy support and thwarting relate to student motivation and academic functioning. *Front. Educ.* 8:1153647. 10.3389/feduc.2023.1153647

[B36] Karimi S. Sotoodeh B. (2020). The mediating role of intrinsic motivation in the relationship between basic psychological needs satisfaction and academic engagement in agriculture students. *Teach. Higher Educ.* 25, 959–975. 10.1080/13562517.2019.1623775

[B37] Lam S. F. Jimerson S. Kikas E. Cefai C. Veiga F. H. Nelson B. et al. (2012). Do girls and boys perceive themselves as equally engaged in school? The results of an international study from 12 countries. *J. Sch. Psychol.* 50, 77–94. 10.1016/j.jsp.2011.07.004 22386079

[B38] Liu G. Teng X. Zhu D. (2019). Effect of self-esteem and parents’psychological control on the relationship between teacher support and chinese migrant children’s academic achievement: A moderated mediation. *Front. Psychol.* 10:2342. 10.3389/fpsyg.2019.02342 31736816 PMC6839426

[B39] Liu J. Bartholomew K. Chung P. (2017). Perceptions of teachers’ interpersonal styles and well-being and ill-being in secondary school physical education students: The role of need satisfaction and need frustration. *Sch. Mental Health* 9, 360–371. 10.1007/s12310-017-9223-6

[B40] Longobardi C. Prino L. E. Marengo D. Settanni M. (2016). Student-Teacher relationships as a protective factor for school adjustment during the transition from middle to high school. *Front. Psychol.* 7:1988. 10.3389/fpsyg.2016.01988 28066305 PMC5179523

[B41] Ma G. (2020). Migrant status, school segregation, and students’ academic achievement in urban china. *Chin. Sociol. Rev.* 52, 319–336. 10.1080/21620555.2019.1680280

[B42] Madjar N. Nave A. Hen S. (2013). Are teachers’ psychological control, autonomy support and autonomy suppression associated with students’ goals?. *Educ. Stud.* 39, 43–55. 10.1080/03055698.2012.667871

[B43] Mammadov S. Schroeder K. (2023). A meta-analytic review of the relationships between autonomy support and positive learning outcomes. *Contemp. Educ. Psychol.* 75:102235. 10.1016/j.cedpsych.2023.102235

[B44] Markkula N. Cabieses B. Lehti V. Uphoff E. Astorga S. Stutzin F. (2018). Use of health services among international migrant children - a systematic review. *Global Health* 14:52. 10.1186/s12992-018-0370-9 29769091 PMC5956827

[B45] Mih V. Mih C. (2013). Perceived autonomy-supportive teaching, academic self-perceptions and engagement in learning: Toward a process model of academic achievement. *Cogn. Brain Behav. Interdiscipl. J.* 17, 289–313.

[B46] Moreno-Murcia J. A. Huéscar Hernández E. Ruiz L. (2018). Perceptions of controlling teaching behaviors and the effects on the motivation and behavior of high school physical education students. *Int. J. Environ. Res. Public Health* 15:2288. 10.3390/ijerph15102288 30340369 PMC6209922

[B47] Ni S. Chui C. H. Ji X. Jordan L. Chan C. L. (2016). Subjective well-being amongst migrant children in China: Unravelling the roles of social support and identity integration. *Child Care Health Dev.* 42, 750–758. 10.1111/cch.12370 27349854

[B48] Opdenakker M. C. (2021). Need-Supportive and need-thwarting teacher behavior: Their importance to boys’and girls’academic engagement and procrastination behavior. *Front. Psychol.* 12:628064. 10.3389/fpsyg.2021.628064 33776849 PMC7988229

[B49] Patrick H. Ryan A. M. Kaplan A. (2007). Early adolescents’perceptions of the classroom social environment, motivational beliefs, and engagement. *J. Educ. Psychol.* 99, 83–98. 10.1037/0022-0663.99.1.83

[B50] Perera K. D. R. L. J. (2022). Application of SDT to promote students motivation and engagement in learning: Non-asian and asian contexts. *Asian Rev. Soc. Sci.* 11, 17–21. 10.51983/arss-2022.11.1.2935

[B51] Pintrich P. R. Smith D. A. F. Garcia T. Mckeachie W. J. (1993). Reliability and predictive validity of the motivated strategies for learning questionnaire (mslq). *Educ. Psychol. Measurement* 53, 801–813. 10.1177/0013164493053003024

[B52] Popp P. A. Grant L. W. Stronge J. H. (2011). Effective teachers for at-risk or highly mobile students: What are the dispositions and behaviors of award-winning teachers? *J. Educ. Stud. Placed Risk (JESPAR)* 16, 275–291. 10.1080/10824669.2011.610236

[B53] Reeve J. (2009). Why teachers adopt a controlling motivating style toward students and how they can become more autonomy supportive. *Educ. Psychol.* 44, 159–175. 10.1080/00461520903028990

[B54] Remedios R. Richardson J. T. (2013). Achievement goals in adult learners: Evidence from distance education. *Br. J. Educ. Psychol.* 83, 664–685. 10.1111/bjep.12001 24175688

[B55] Ryan R. M. Deci E. L. (2020). Intrinsic and extrinsic motivation from a self-determination theory perspective: Definitions, theory, practices, and future directions. *Contemp. Educ. Psychol.* 61:101860. 10.1016/j.cedpsych.2020.101860

[B56] Shih S. (2008). The relation of self-determination and achievement goals to taiwanese eighth graders’behavioral and emotional engagement in schoolwork. *Elemen. Sch. J.* 108, 313–334. 10.1086/528974 40917494 PMC12412616

[B57] Soenens B. Sierens E. Vansteenkiste M. Dochy F. Goossens L. (2012). Psychologically controlling teaching: Examining outcomes, antecedents, and mediators. *J. Educ. Psychol.* 104, 108–120. 10.1037/a0025742

[B58] Van Yperen N. W. Blaga M. Postmes T. A. (2015). meta-analysis of the impact of situationally induced achievement goals on task performance. *Hum. Perform.* 28, 165–182. 10.1080/08959285.2015.1006772

[B59] Vansteenkiste M. Zhou M. Lens W. Soenens B. (2005). Experiences of autonomy and control among chinese learners: Vitalizing or immobilizing? *J. Educ. Psychol.* 97, 468–483. 10.1037/0022-0663.97.3.468

[B60] Wang J. Xing Q. Moè A. (2024). Understanding the dynamics of teaching styles and need satisfaction in the chinese educational context. *Teach. Teach. Educ.* 145:104609. 10.1016/j.tate.2024.104609

[B61] Williams G. C. Deci E. L. (1996). Internalization of biopsychosocial values by medical students: A test of self-determination theory. *J. Pers. Soc. Psychol.* 70, 767–779. 10.1037//0022-3514.70.4.767 8636897

[B62] Yang Hansen K. Radišić J. Ding Y. Liu X. (2022). Contextual effects on students’ achievement and academic self-concept in the nordic and chinese educational systems. *Large-scale Assess. Educ.* 10:16. 10.1186/s40536-022-00133-9

[B63] Yu J. Kreijkes P. Salmela-Aro K. (2023). Interconnected trajectories of achievement goals, academic achievement, and well-being: Insights from an expanded goal framework. *Learn. Individ. Differ.* 108:102384. 10.1016/j.lindif.2023.102384

[B64] Zhang L. Feng Z. (2025). The mediating role of teacher support in the relationship between marginalization and academic performance among migrant children in china. *J. Global Trends Soc. Sci.* 2, 107–116. 10.70731/3bvnpc94

[B65] Zhang L. He G. Chen Y. Shi A. (2020). Migration status, emotional engagement, and social exclusion in Chinese schools. *J. Adolesc.* 80, 192–203. 10.1016/j.adolescence.2020.02.013 32169719

